# From Lateral Flow Devices to a Novel Nano-Color Microfluidic Assay

**DOI:** 10.3390/s90806084

**Published:** 2009-07-31

**Authors:** Saied Assadollahi, Christiane Reininger, Roland Palkovits, Peter Pointl, Thomas Schalkhammer

**Affiliations:** 1 Attophotonics Biosciences GmbH, Viktor Kaplan Strasse 2, A-2700 Wiener Neustadt, Austria; E-Mails: palkovits@attophotonics.com (R.P.); Reininger@attophotonics.com(C.R.); pointl@attophotonics.com (P.P.); 2 Max F. Perutz Laboratories, Department of Biochemistry and Molecular Cell Biology, University of Vienna, A-1030 Vienna, Austria; E-Mail: saied.assadollahi@univie.ac.at (S.A.); 3 Departments of Micro and Nanosystems, University of Applied Sciences, A-2700 Wiener Neustadt, Austria; E-Mail: roland.palkovits@fhwn.ac.at (R.P.)

**Keywords:** lateral flow assays (LFAs), resonance-enhanced absorption (REA), AuNPs-bioconjugate, polyethylene terephthalate (PET), CO_2_ laser ablation

## Abstract

Improving the performance of traditional diagnostic lateral flow assays combined with new manufacturing technologies is a primary goal in the research and development plans of diagnostic companies. Taking into consideration the components of lateral flow diagnostic test kits; innovation can include modification of labels, materials and device design. In recent years, Resonance-Enhanced Absorption (REA) of metal nano-particles has shown excellent applicability in bio-sensing for the detection of a variety of bio-molecular binding interactions. In a novel approach, we have now integrated REA-assays in a diagnostic microfluidic setup thus resolving the bottleneck of long incubation times inherent in previously existing REA-assays and simultaneously integrated automated fabrication techniques for diagnostics manufacture. Due to the roller-coating based technology and chemical resistance, we used PET-co-polyester as a substrate and a CO_2_ laser ablation system as a fast, highly precise and contactless alternative to classical micro-milling. It was possible to detect biological binding within three minutes – visible to the eye as colored text readout within the REA-fluidic device. A two-minute *in-situ* silver enhancement was able to enhance the resonant color additionally, if required.

## 1. Introduction

**Figure 1.**
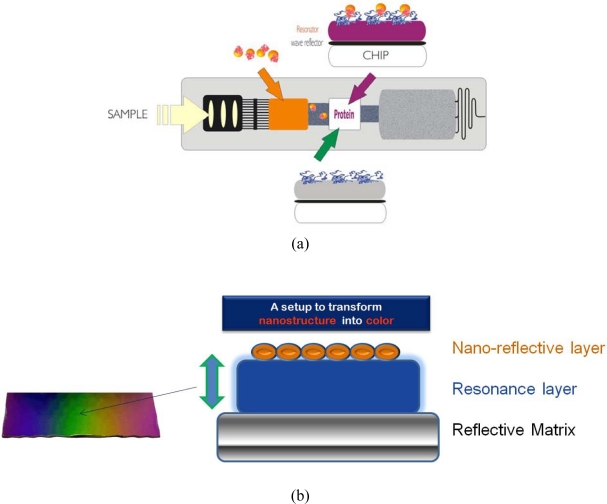
(a) Design and setup of resonance-color fluidic test sticks for proteins and DNA. (b) Scheme and setup of resonance-color surfaces.

A novel nanocolor based assay [Figure 1(a)] was developed for analysis of urine and blood samples and integrated into a fluidic cartridge to replace lateral flow assays (LFAs), thus resolving fundamental drawbacks of metal-nano-particle based assays and devices [1–6].

Metal clusters transduce molecular binding events with high sensitivity based on their extraordinary extinction coefficients. In a REA-set-up those clusters [as a nano-granular reflective layer, see Figure 1(b)] give rise to a color visible to the eye if bound via biomolecules at a resonance-distance to the mirror. This distance of strong coloring starts at around 20 nm nanometer from the matrix reaching out to around 300 nm. The color is dependent on the thickness of the resonance layer, the number of clusters as well as the angle of observation [7,8].

LFAs are still the primary means for the detection of a variety of analytes including pathogens and toxins [9–11]. Individual assay layouts are adapted to the particular application. Nevertheless, some basic features are common to most of the systems that are commercially available. Typically, a ligand such as an antibody is immobilized in a testing zone on a porous membrane such as nitrocellulose. Antibodies labeled with colloidal gold, carbon black, dyed latex particles or fluorescent markers are often used as reporter molecules [12,13]. These are either separately mixed with the sample or directly bound to the conjugate pad which is attached to the membrane. In addition, the conjugate pad contains buffer chemicals, blocking reagents and stabilizers. A sample-pad above the conjugate-pad pre-filters the sample and an absorbent-pad takes up the liquid on the opposite side of the membrane in the assay setup. When a sample such as blood or urine is added, the fluid migrates by capillary force toward the absorbent pad. The analyte/reporter complex binds to the test zone and excess reagents and fluid are transported to the absorbent pad. In most assays, the fluid passes through a control zone, where suitable ligands capture excess reporter molecules ensuring that the sample fluid migrated through the test. The test result is read-out either with the naked eye and/or quantified via an optical scanner.

**Figure 2.**
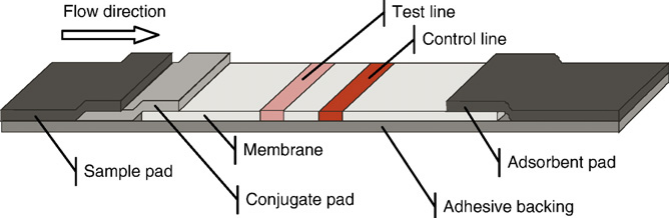
Standard layout of a lateral flow device.

Figure 2 gives a schematic view of a typical LFA. A porous membrane is mounted on an adhesive backing. A specific ligand such as an antibody is immobilized on the test line. On the control line, capture ligands for the reporter molecules are bound. The sample and conjugate pads enable the equal distribution of the liquid sample and serve as a reservoir for the assay reagents. The adsorbent pad soaks up excess liquid and enables sufficient flow-through [14].

Lateral flow tests [15], also known as lateral flow immune-chromatographic assays, are an extension of latex agglutination tests which were developed in 1956 by Singer and Plotz [16]. Most of these tests are commonly used in point-of-care (POC) devices for medical diagnostics. Lateral flow assays are widely used for the qualitative detection of several analytes in pregnancy tests (β-chorionic gonadotropin), fertility and ovulation tests (lutinizing hormone and follicle stimulating hormone), infectious diseases, cancer diagnostics, cardiac or drug abuse markers (amphetamines, cocaine and benzodiazipanes) sold by companies such as Unipath, Abbott, or Quidel.

Due to the materials, labels, and device design used in traditional LFAs, there are some inherent weaknesses in the functions of these assays. Nitrocellulose, glass fiber, polyester, rayon and other filtration membranes have all been adapted for use in these assays. Nitrocellulose is the most widely used membrane, but is limited by unspecific binding and a highly flammable matrix. Furthermore, shelf-life, brittleness and relative sensitivity to humidity are critical [17].

Typically gold nanoparticles, colored or fluorescent latex beads dried onto conjugate pads are used as labeling agents, resulting in high levels of variation due to instability and inconsistent release. Additionally, in a three-dimensional matrix such as nitrocellulose, the signal is obtained from only approximately the top 10 μm of the membrane (thickness: 100–200 μm) and the remaining signal in the system is lost [18]. Novel markers, coupled with alternative materials and reading systems, can yield consequential improvements in performance and could be alternative approaches to the use of existing labels.

### 1.1. Design of Microfluidic Systems

It was essential to develop innovative designs that retain desirable features of the traditional LFA, but avoid the major associated problems. This applies to the entire assay device, not just to the membrane system. Although some attempts to innovate have been made over the past 10 years, truly novel approaches to LFA materials, particularly reaction surfaces and conjugate application pads, have not been numerous [18].

In addition to the technical restrictions for LFAs, a combination of other factors has driven a renaissance in lateral flow technologies which include patent pressures on existing technologies; new technologies with increased sensitivity, higher reproducibility and quantitative or qualitative analysis. Demands for appropriate POC or field-use technologies can be brought to market in a short time frame and for a reasonable investment [17]. In this context microfluidics have an acknowledged position in the developmental process of LFAs, and represent a technology that complements and will eventually supplant LFAs.

Microfluidic systems provide a powerful platform for biological assays [19–21]. In microfluidic devices, small volumes of liquids are moved through micro-channels generated in a cartridge or chip. Bioassays, which have been miniaturized into a chip format, are obtainable for DNA sequencing, polymerase chain reaction (PCR), electrophoresis, DNA separation, enzymatic assays, immunoassays, cell counting, cell sorting and cell culture [22–24]. Nevertheless, microfluidic devices have some limitations:

- Mixing of liquids can only be performed by diffusion due to laminar flows: if mixing is required, channels need to be of sufficient length to allow an acceptable flow rate.
- Large capillary forces: small channels lead to large capillary forces, which may drive fluids into unwanted areas of the device risking contamination of other fluid streams.
- Possible evaporation: st small scales, the surface to volume ratio is large. Thus, fluids exposed to the surroundings rapidly evaporate.
- Clogging of channels: small structures cause bottlenecks and even blocking of channels.

To fully utilize the advantages of fluidics, it is advisable to design fluidic devices with optimized channel dimensions and geometry preventing desiccation of the system. Due to the special arrangement of the channels and reservoirs, blocking of liquid flow is also minimized. We describe below a novel layout for fluidic assays using REA effects which can be adapted for application in many POC diagnostics.

### 1.2. Manufacturing Methods of (Micro)Fluidic Devices

**Figure 3.**
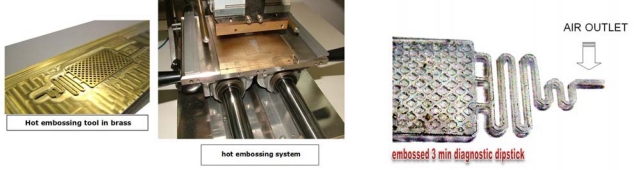
Hot embossing unit, tool and hot embossed air outlet structure to prevent leakage of fluid from the REA-device.

Methods described for the generation of channels and reservoirs in (micro)fluidic devices in substrates such as glass and silica include photolithography or mechanical and laser ablation. For thermoplastics, techniques such as soft-lithography of PDMS (polydimethylsiloxane), microinjection molding, hot embossing (Figure 3) and microfluidic tectonics are used. Recently, a novel contact liquid photolithographic polymerization (CLIPP) technology was presented by K. Haradsson [25]. Thermoplastic materials such as poly[methyl methacrylate] (PMMA) and co-polyesters have been intensively investigated and used for generating microfluidic devices by industrial laser ablation [26–29].

“Resonance Enhanced Absorption” (REA) of bio-conjugated metal nanoparticles was developed in our lab for detection of a variety of bio-analytes and applied by other research groups [7,8]. Although the REA-method has a number of advantages compared to traditional assays, the most critical drawback that hindered application of this assay for POC-diagnostics is a rather long incubation time. In this paper we describe the implementation of a REA-assay in a microfluidic cartridge to substantially shorten the incubation time as a major breakthrough in REA-application in diagnostics.

## 2. Experimental Section

### 2.1. Materials

Bovine IgG, anti-bovine IgG from rabbit, human serum albumin (HSA), fractionated anti-albumin goat antiserum, aqueous polyethyleneimine (PEI) solution 50% (w/v), poly(4-styrenesulfonic acid-*co*-maleic acid) sodium salt, palladium chloride, hydroquinone, Tween 20, lipase, polyethylene glycol (PEG) MW 4,000, trisodium citrate and PMMA were obtained from Sigma-Aldrich (St. Louis, MO, USA). Silver nitrate, l-ascorbic acid and glycerin were purchased from ACM (Herba-Chemosan, Vienna, Austria) and tetrachloroauric acid [III] trihydrate from Merck GmbH (Vienna, Austria). Aqueous 25% (v/v) glutaraldehyde solution and microscope slides were obtained from Menzel GmbH + CoKG (Braunschweig, Germany), aluminized polyethylene terephthalate (Alu-PET) films from Hueck (Baumgartenberg, Austria) and temperature-proof adhesive tapes No. 4910F from Tesa^®^ (Hamburg, Germany). *Vivak* co-polyester sheets were purchased from Bayer Sheet Europe (Darmstadt, Germany) and polyamide porous membranes from Eckert (Germany). Commercial ELISA kits (BMS213/2MST) for interleukin-6 (IL-6) were obtained from Bender Medsystems (Vienna, Austria).

### 2.2. Equipment

BOC Edwards Auto 306 (oil diffusion pumped) for physical vapor deposition (PVD), CO_2_ laser engraver (10.6 μm, 30 W), Attophotonics spin coater and Attophotonics turbomolecular pumped sputter coating system, fiber optics spectrophotometer for recording assay kinetics and a corona plasma unit for surface hydrophilization.

### 2.3. Bioconjugate Preparation

Gold nano-particles (AuNPs) were prepared according to Frens *et al*. [30]. Bovine IgG (30 μL) was dialyzed against distilled water and added to 45 mL of pH adjusted AuNPs under constant stirring. After 30 min, the conjugate was stabilized by the addition of 2.55 mL of 10% PEG solution. The conjugate was concentrated 12 to 64 fold by centrifugation and stabilized by the addition of lipase. A highly concentrated salt or buffer solution (e.g. lactate) was added to the concentrated gold conjugate and mixed thoroughly. The detail procedure for preparation of AuNPs and the bioconjugate has been described in a previous paper [31].

### 2.4. Prototyping and Testing of the Fluidic Device

The flow-through cell was designed as follows: two strips of PET film (8 mm × 60 mm and a thickness of 25 μm) were fixed on a glass slide (via glue) in such a manner that a channel of 10 mm width, 60 mm length and approximately 40 μm depth (thickness including the glue) was created. As a wicking pad, a rigid and highly absorptive paper was attached on the free end of the channel.

For preparation of the REA-chips, two different methods were used. Glass slides were either metalized with aluminum by physical vapor deposition followed by SiO_2_-coating. The average thickness of the aluminum film was 90 nm with 250 nm of SiO_2_. These coated glass slides (REA-chips) were cut into 12.5 mm × 12.5 mm pieces. Alternatively, an aluminized PET-film was coated with organic and inorganic polymers via spin-coating and PVD. They were also divided into 12.5 mm × 12.5 mm pieces and mounted over a glass slide using a double sided Tesa^®^ adhesive tape with the same dimensions. The REA-chips were then modified with PEI followed by glutaraldehyde activation and incubation with anti-bovine IgG. The chips were incubated in a humid chamber at RT for 15 min and subsequently washed, blocked and rewashed.

## 3. Multi-Step Development of the Fluidic Device, Results and Discussion

### 3.1. Binding Kinetics

The flow-through cell described above was placed on the top of a specially adapted fiber optics spectrophotometer and the REA-chip was positioned on it with the REA-side below, adjacent to the wicking pad. Due to this set-up, a micro-channel was formed and the illuminated light could irradiate to the site of immobilized anti-bovine IgG on the REA chip. A solution containing the AuNPs-IgG conjugate was transferred to the cell and laminar flow passed under the REA chip. The AuNPs-bioconjugate was bound to the REA chip via antigen-antibody interaction and the excess liquid was absorbed to the filter paper.

The kinetics of the reaction between the immobilized anti-bovine IgG and AuNPs-IgG was recorded for five minutes.

To test performance and feasibility of the fluidic device another antigen-antibody couple, HSA/anti-HSA was conjugated to 33 nm AuNPs followed by testing the conjugate in a sandwich ELISA setup. 10 μL of the 1:100 dilution of anti-HSA in 1×PBS were dotted on the activated REA-chips for 10 min in a humid chamber at RT followed by washing, blocking and rewashing. 10 μL of increasing concentrations of HSA (from 1 μg/mL to 1 mg/mL) were then dotted on the REA chips for 2 min. As negative controls, 1×PBS and 100 μg/mL lipase were dotted. After two minutes, all chips were washed and placed one by one on the fluidic cell and 5× concentrated anti-HSA-AuNPs conjugate was added to the fluidic cell and the kinetics of the reaction recorded by the fiber photometer.

Due to the weak optical signal on the chips, the REA-effect was enhanced by silver staining. 10 μL of 45 mg/mL silver nitrate was added to 350 μL of 150 mM citrate buffer and mixed thoroughly. 75 μL of 2.5% freshly prepared hydroquinone was then added to the solution and REA-chips with bound AuNPs were placed on the fluidic cell again. The silver enhancement solution was added to the cell and the kinetics of the reaction was recorded for two minutes. Using higher concentrations of AuNPs-anti HSA (32–64 fold concentrated), vivid resonant colors visible to the eye were obtained.

In our prototype fluidic setup, we immobilized and dried AuNPs-anti HSA similar to commercially available standard lateral flow assay devices. 20 μL of 40× concentrated AuNPs conjugate was therefore mixed with 20 μL of 5% sucrose and dotted on the fluidic cell 2 cm from the position of the REA chip. After drying of the gold conjugate on the fluidic cell, a REA chip immobilized HSA was placed on the fluidic cell and a 1:3 dilution of pH = 7 lactate buffer was added to the cell. Dried AuNPs-anti HSA were dissolved and then absorbed to the wicking pad. After 3–5 min, the test was stopped and a REA-color dot was visualized on the chip. To increase the local gold concentration, the gold bioconjugate was dried in a Petri dish and then agitated free and placed in the fluidic cell as before. Following the addition of a 1:3 dilution of lactate buffer, a gold bioconjugate suspension was formed more quickly and consequently a more intense REA-color was generated.

The applicability of the fluidic nanoparticles binding assay was also tested for detection of an important cytokine (Interleukin-6) of the human plasma. The abnormal production of IL-6 was first suggested to be related with cardiac myxoma [32] and then pathogenesis of a variety of diseases.

To setup a nanoparticle binding assay for detection of IL-6, a set of antibodies and antigens identical to the ones employed in the ELISA kit (containing coating antibody (100 μg/mL), IL-6 standard protein (2 ng/mL after reconstitution), biotin conjugate, horse radish peroxidase (HRP) conjugated Streptavidin and assay buffer) was used.

AuNPs (10 mL of a 33 nm particle suspension) were adjusted to pH 8.0 and 6 μL of Streptavidin-HRP was injected into the AuNPs solution under constant stirring which was continued for 1 hour. The AuNPs-conjugate was then stabilized by the addition of 10% PEG (Mw = 4,000). The final concentration of PEG in the nanoparticle conjugate was 0.5%. Four REA-chips were then surface-modified and 5 μL of coating antibody were dotted onto the chips in a humid chamber for 30 min. The chips were washed with washing buffers and blocked with 1% lipase in 1×PBS, 0.5% Tween 20. After a second wash, 20 μL of IL-6 (2 ng/mL) was loaded onto three REA-chips and incubated overnight at 4 °C in a humid chamber. The fourth chip served as a negative control and was left in a humid chamber without incubation with IL-6. The next day, three test chips were washed with washing buffers and 30 μL of 1:10, 1:20 and 1:30 dilutions of biotin-conjugate in assay buffer were incubated on the test chips. On the negative control chip, the mean value (1:20 dilution of biotin-conjugate) was used. After 1 hour incubation in a humid chamber at RT, the washing step was repeated for all chips. Then 100 μL of a 20× concentration of AuNPs-streptavidin-HRP conjugate was used in a flow-through setup and the AuNPs-conjugate was captured on the REA chip. Finally the silver enhancement procedure was performed on all chips.

### 3.2. Palladium Nanoparticles (PdNPs) as an Alternative to AuNPs in Binding Assays

Up to now, AuNPs have been widely used both in resonant and non-resonant binding capture assays. Recently, palladium-coated gold nanoparticles were used as Surface-Enhanced Raman Scattering substrates [33] and production of hydrogen peroxide [34]. PdNPs are noble nanoparticles that have some advantages for usage in rapid tests. Palladium is about three times less costly than gold and palladium nanoparticles are also much more efficient as catalysts in the silver enhancement process. In addition, preparation of palladium nanoparticles is easy and can be performed without heating the palladium salt solution to the boiling point. After stabilization with polymers, these nanoparticles also show rather good stability over long periods of time. PdNPs were therefore prepared and used in a fluidic bioassay.

Ascorbic acid (2.5 g) and 100 mg of poly(4-styrene sulfonic acid-*co*-maleic acid) sodium salt (Mw = 20,000) were dissolved in 48 mL of double distilled water (dd H_2_O) and incubated at 60 °C under vigorous stirring. One mL of 2% PdCl_2_ was then injected into the solution and stirred continuously for 10 min. The PdNP preparation was then transferred into microcentrifuge tubes and after centrifugation at 10,000 rpm for 10 minutes, PdNP pellets were obtained. The pellets were resuspended in 50 mL of ddH_2_O and the original concentration was recreated. To prepare palladium bioconjugate, 15 mL of purified PdNPs were diluted three times by the addition of 30 mL ddH_2_O. The pH was then adjusted to 8.0–8.5 using 1 M of Na_2_CO_3_. Forty five μL of anti HSA solution was then diluted in 1 mL of ddH_2_O and injected into the PdNPs during stirring which was continued for 30 minutes at RT.

The surface of one REA-chip was activated as described above and was dotted with 10 μL of 20 μg/mL HSA on the chip. After 15 minutes of incubation in a humid chamber at RT, the REA-chip was washed, blocked and rewashed. Finally, PdNPs-anti HSA conjugate was concentrated 20× by centrifugation and then 53.5 μL of the concentrate was mixed with 10 μL of 10 mg/mL Lipase in 1×PBS and incubated for 30 min. The REA-chip with immobilized HSA was then placed on a fluidic cell and 36.5 μL of lactate buffer pH 7.0 was mixed with stabilized PdNPs-anti HSA conjugate and loaded into the channel of the fluidic cell. The binding reaction was continued for 3 minutes and the REA-chip was washed with ddH_2_O and air dried. It was possible to obtain a resonant color signal over the REA-chip that was a consequence of bound PdNPs in nanometric distance of the REA-chip.

In our previous setup, we used silver enhancement for amplifying the resonant signal of AuNPs. The same procedure was repeated using bound PdNPs on the REA chip. Unlike AuNPs, that needed two minutes to enhance the resonant signal, PdNPs were able to catalyze the process on the flow-through cell within 5–10 seconds.

**Figure 6.**
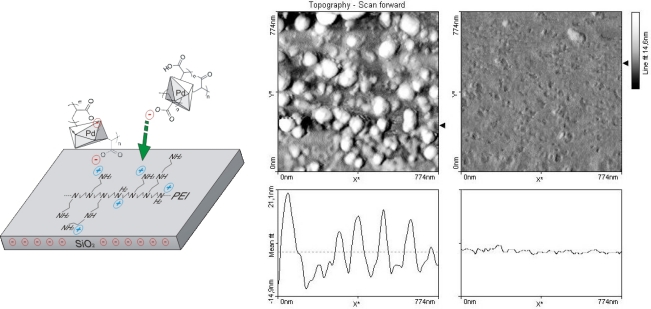
Coating and AFM scan of palladium nanoparticles (used as catalytic seeds in the fluidic device).

The size-distribution of the PdNPs was analyzed via Atomic Force Microscopy (Figure 6). A glass slide was sequentially washed with 3% HNO_3_, 10% NaOH and 2-propanol. Following each wash, ddH_2_O was use to rinse the slide. The slide was then incubated in 1% PEI Mw = 1,200 for 30 min under constant stirring, washed with ddH_2_O and dried with compressed-air. 10μL of 20× concentrated PdNPs were incubated on the glass slide overnight in a humid chamber. The glass slide was then washed in a large volume of ddH_2_O and dried. The area of glass slide with bound PEI and PEI-PdNPs were then scanned by AFM. The size of prepared PdNPs was determined to be approximately 20 nm with a subpopulation of 10–15 nm in diameter.

After confirmation of the basic principles of the REA-assay in a simple fluidic device, our attempts was focused on designing a more elaborate microfluidic cell.

### 3.3. Designing and Fabrication of the Microfluidic Cell

As a fast, highly precise and contactless alternative to mechanical milling, we used a CO_2_ laser ablation system for creating microstructures in co-polyester films (PET) with a thickness of 0.6 mm. A PC-based control program was used as scheduler to set the laser power and the laser scanning speed.

**Figure 7.**
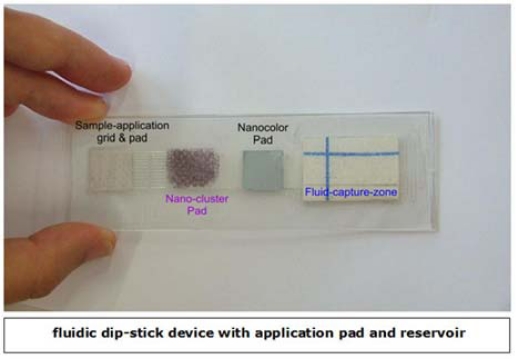
Setup of the laminated – laser-ablated fluidic device.

In the first few tests a simple fluidic pattern was designed on the PET sheets with the following laser machining parameter settings: resolution 300 dpi, power intensity 100%, speed of 50 mm/s, offset (focus) 0.1 and 3 cycles of laser ablation. Due to the strong hydrophobicity of the PET sheets the surface of the fluidic cell was exposed to a 17 kV high-voltage corona pre-treatment causing a hydrophilization of the surface. The PET sheet with the microfluidic structures was covered with a thin adhesive tape and the flow of a tracer dye (Neutral Red) was investigated. In another setup a resolution of 600 dpi and structuring parameters of 85/65/0.1/2× (parameter-sequence as above) were used. After several modifications, a setup with resolution of 500 dpi and settings of 70/55/0.1/3× showed optimal results (Figure 7).

In the next step, flow characteristics in the vertical position, which are dependent on gravity and capillary forces between comb-like structures and parallel channels, were examined: parallel micro-channels exhibited stronger capillary forces than comb-like structures however, the capillary forces were not strong enough and the tracer dye stopped in the middle of the fluidic pattern. Nevertheless, the comb-like structure had a better capacity in collecting the end-flow than parallel channels.

Since it was found that the fluidic device could not be used in a vertical position like dip-sticks, attempts were focused on designing a lateral fluidic device. In a fabricated fluidic device several holes in the PET film were used as the sampling site. Additionally engraved parallel channels and comb-like structures were employed for conducting the flow and collecting the end-flow.

**Figure 8.**
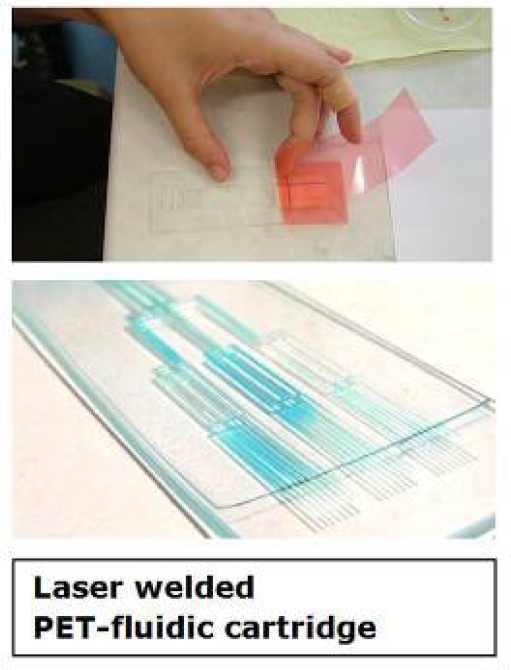
Laser-ablated fluidic device.

Another critical feature of the fluidic cell was the leakage of the flow from the end point of the collecting pad. During testing of the fluidic device, it was observed that the tracer dye reached the end of the device and exuded to the outside. With respect to this problem, corona pre-treatment of the reservoir section on the fluidic cell was avoided and the fluid was halted in the reservoir. One of the key problems in laser engraving of micro-capillaries was removal of the thermal damage-associated debris which could block the channels. Washing with ethanol and compressed-air was examined for removal of the debris. Although this method could resolve blockage of the channels, ethanol rinsing following laser ablation converted laser-ablated parts of the PET sheets to a turbid appearance. By replacing ethanol with dH_2_O in the above method, the occurrence of a turbid surface layer was avoided (Figure 8).

As mentioned previously, the prototypic fluidic device was composed of a PET sheet coated with a thin adhesive film. Therefore, the whole structure did not have the desired stability and robustness required for handling. To improve mechanical stability, another PET sheet was used to replace the thin adhesive tape and was fixed with liquid glue over the laser-ablated PET film. Despite this modification, the new setup was not durable against tension and traction and the layers were prone to detach. Moreover, the liquid glue penetrated into the channels causing blockage. A double-sided adhesive film replacing the organic solvent-based glue enhanced stability of the fluidic cell and no further blockage of the channels was observed.

In the next step similar to the commercial LFAs, AuNPs-bioconjugate was dried in one of the void structures on the fluidic cell. The supporting PET film was fixed over the cell and diluted buffer or dH_2_O was loaded on the sampling site. The liquid reached the dried AuNPs-bioconjugate, but could not re-suspend the dried colloidal gold. A change in design pattern of channels and creation of larger cavities could not resolve this problem. In some cases, small particles of dried AuNPs-bioconjugate were detached from the main dried mass and blocked the channels. Instead of drying the AuNPs-bioconjugate directly in cavities, it was dried on a piece of open pore filter paper. On addition of dH_2_O, the AuNPs-bioconjugate was released from the paper, flowed into the channel, passed over the REA-chip and was taken up by the absorbent reservoir. Although the channels did not block, most of the gold could not be released from the paper and remained absorbed.

To improve the release of the AuNPs-bioconjugate from the conjugate pad, a double-layer fleece of polypropylene was cut to the size of the conjugate cavity, washed with ethanol and dried. Due to the hydrophobicity of the polypropylene fleece, the AuNPs-bioconjugate (mixed in a 1:1 ratio with 5% sucrose) could not be adsorbed onto the conjugate pad. Tween 20 was therefore added to the AuNPs –bioconjugate before adding to the fleece and subsequent drying. When a 1:3 dilution of lactate buffer was added to the sample pad, almost all of the gold conjugate was released from the conjugate pad and contacted the end of the fluidic cell.

Another major disadvantage for this setup was the application of a 17 kV corona for surface hydrophilization of the fluidic cell. Although corona treatment could enhance the hydrophilicity of the PET sheet, the effect was temporary and after a few days, the PET sheet returned to a hydrophobic state. As a permanent solution to this problem, a solution of 1.5% PVP was prepared in ethanol and the ablated parts of the fluidic cell were painted once with this solution. PVP-coated fluidic cells were used in several tests and all showed high hydrophilicity in channels and cavities.

### 3.4. Optimization of the Fluidic Cell

In the previous set-up, it was found that comb-like structures were suitable as cavities for collecting end-flow and parallel micro-channels for conductance of the fluid. In this assembly, we tried to achieve a functional layout for a microfluidic device with optimized flow conduction in channels and cavities. Our fluidic cell had been composed of two identical-sized PET sheets with the dimensions of 120 mm × 35 mm and a thickness of 0.6 mm. One of these sheets functioned purely as a support and the other was used for designing the fluidic layout.

Since the fluidic device was fabricated as a lateral flow fluidic device, the sample pad was composed of several holes in the PET film and fluidic samples could be loaded with a pipette onto the fluidic device from the upper side. The sample pad was composed of nine holes each with a diameter of 2 mm that were spread homogeneously on a pad area of 1.2 cm × 1.2 cm. Due to diminutive capillary forces in the very short channels, the distance between the sample pad and conjugate pad was set to 8–10 mm. A smaller distance led to a partial release of reagents from the conjugate pad. Furthermore, parallel channels exhibited a better performance in releasing the reagents from the conjugate pad than a combination of channel and comb-like structures. Across the width of the fluidic cell, fourteen parallel channels were deployed at the distance between sampling and conjugate pad. These channels were also connected by short channels for synchronization of the flow channels. Whereas the width of the REA-chip was less than the conjugate pad, the distance between the conjugate pad and the position of the REA-chip was arranged with an array of convergent parallel channels. The area of the REA-chip and collecting pad were connected to each other through three parallel channels.

Previously, we examined the effect of Tween 20 in a ‘low binding energy’ adsorption of AuNPs-bioconjugate to the fleece pad. This detergent was also beneficial in releasing the AuNPs-bioconjugate from the fleece pad. In an experiment, AuNPs-bioconjugate was dried with and without Tween 20 on two bioconjugate pads and the rates of particles release was examined. It was observed that Tween 20 helps extensively to release the AuNPs-bioconjugate from the conjugate pad.

**Figure 9.**
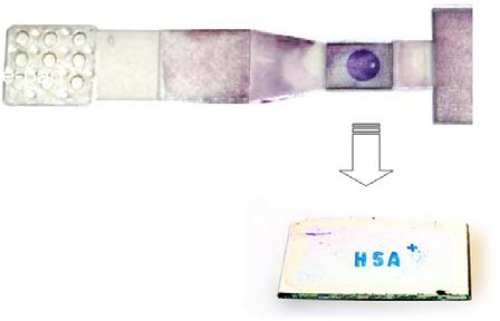
Optimized layout of laser-ablated fluidic device with REA-chip and Text-output via the nanocolor-antibody assay.

Two more strategies were employed to improve efficient release of the AuNPs-bioconjugate from the conjugate pad and smooth running of liquid in the conducting channels. Initially another piece of polypropylene fleece pad was coated with a 1:1 mixture of 2% Tween 20 and 5% sucrose and then lodged in the sample cavity. The second strategy was to increase the volume of the collecting pad and its suction efficiency by creating a hollow reservoir with spongy pads as the absorbent substrate. In this context, spongy rayon pads and a tissue composed of 1/3 cotton and 2/3 cellulose as absorbing textiles were tested. Several tests were also carried out using both as the absorbent pad. The combination of cotton and cellulose showed much better performance in absorption of the fluid from the conducting channels and was capable of absorbing up to 600 μL of the liquid sample, more than double that achieved with the collecting chamber with a comb-like structure. In a comparison test between the new and old absorbing system, it was shown that the release of AuNPs-bioconjugate from the conjugation pad and the stronger suction in the collecting chamber positively influenced the intensity of the resonant dot on the REA-chip (Figure 9).

As utilized in the prototype flow-through cell, silver staining was performed similarly in this new fluidic device. In an experiment, a REA chip was immobilized with HSA and assembled in the fluidic device. After addition of 250–300 μL lactate buffer to the fluidic device, without delay 200 μL of the silver enhancement solution was loaded onto the sampling pad. Consequently, the intensity of the resonant color dot was increased significantly.

Another modification to improve the fluidic device was the generation of a chaotic flow over the REA chip in the fluidic device structure. Due to laminar flow in the micro-channels, no turbulence arose in the channels and the striped pattern of flow was emulated over the resonant color dot. To avoid this effect, a nylon grid was utilized to make a uniform flow over the REA chip. In this context, nylon grid PA100/49 and PA64/45 were tested over the REA chip. The nylon grid with a larger pore size (PA100/49) had better results in creating a chaotic flow over the REA chip and the REA signal was no longer striped. Nevertheless, the nylon grid was to some extent opaque and in commercial production a transparent grid should be employed.

## 4. Conclusions

**Figure 10.**
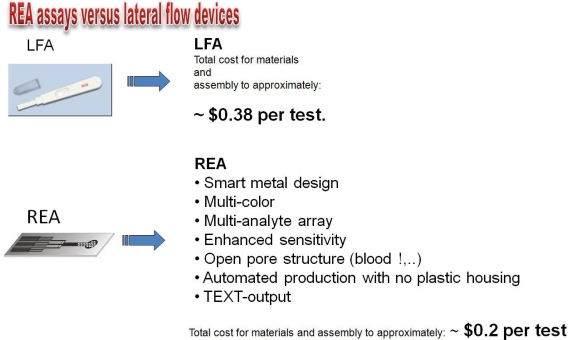
Comparison of features: LFA compared to resonant nanocolor quick test.

**Figure 11.**
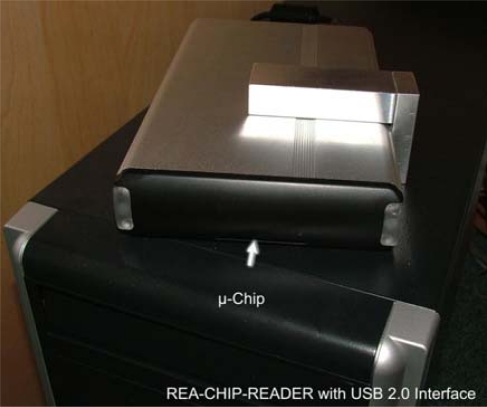
Novel camera-based reader for readout and quantification of the assay.

A significant number of approaches were taken over the last decades to overcome some of the inherent weaknesses of traditional LFAs (Figure 10). Using nano-color technology as an innovative and rapidly developing approach in the area of molecular analysis, we have developed a diagnostic device based on the Resonance-Enhanced Absorption (REA) of metal nanoparticles. This optical thin-film system in chip format is set up on an aluminized PET film coated with organic or inorganic polymer resonance layers. Visualization of the signal is done by metal nanoparticles conjugated to an antibody - binding to the immobilized analyte on the surface of the REA-chip. Compared to the signal in a non-resonant capture assay employed in standard LFA-tests (using AuNPs as well), resonant color diagnostic devices exhibit 7-fold more signal intensity. In contrast to a traditional LFA, the signal is generated as a single (sub) monolayer of metal on the chip-surface. In our novel device, we combined resonant nano-color films, bioanalytical reagents and a fluid-guiding (open pore !) device in one unit manufactured by lamination of PET films to a sandwich. Readout and quantification was performed by a novel camera-based reader (Figure 11).

By implementation of new technologies such as microfluidics, nanocolors and laser ablation, we successfully developed a POC-device that was able to visualize an antibody-based assay within approximately 3 minutes. Enhancement to obtain a strong resonant color on the REA chip was performed by further silver enhancement for around two minutes within the fluidic device. Laser ablation was the method of choice for manufacturing micro-structures for highly specific assays of critical analytes such as interferons or interleukins.

## Figures and Tables

**Figure 4.**
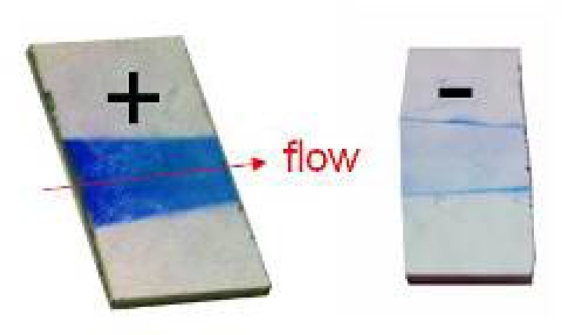
Resonance color obtained by nanoparticle binding in the test system–fluidic cell.

**Figure 5.**
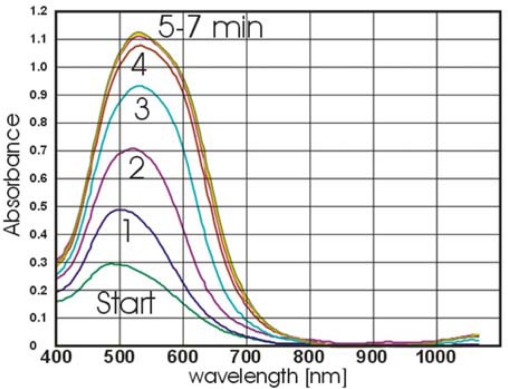
Spectral changes observed by binding of nanoparticles to antibodies deposited as a spot of 5 mm diameter in the device.
